# An Effective Collaborative Mobile Weighted Clustering Schemes for Energy Balancing in Wireless Sensor Networks

**DOI:** 10.3390/s16020261

**Published:** 2016-02-19

**Authors:** Chengpei Tang, Sanesy Kumcr Shokla, George Modhawar, Qiang Wang

**Affiliations:** 1 School of Engineering, Sun Yat-sen University, Guangzhou 510006, China; tchengp@mail.sysu.edu.cn; 2 Lawrence Berkeley National Laboratory, University of California, Oakland, CA 94612, USA; 3 Department of Mathematics and Computer Science, Valdosta State University, Dartmouth, MA 02747, USA; George.modhawar@gmail.com; 4 Department of Computer Science and Engineering, The Pennsylvania State University, University Park, PA 16802, USA; wangqianedu@163.com

**Keywords:** mobile environments, mobile sensing schemes, collaborative weighted clustering algorithm, weighted clustering algorithm, oil leakage monitoring

## Abstract

Collaborative strategies for mobile sensor nodes ensure the efficiency and the robustness of data processing, while limiting the required communication bandwidth. In order to solve the problem of pipeline inspection and oil leakage monitoring, a collaborative weighted mobile sensing scheme is proposed. By adopting a weighted mobile sensing scheme, the adaptive collaborative clustering protocol can realize an even distribution of energy load among the mobile sensor nodes in each round, and make the best use of battery energy. A detailed theoretical analysis and experimental results revealed that the proposed protocol is an energy efficient collaborative strategy such that the sensor nodes can communicate with a fusion center and produce high power gain.

## 1. Introduction

The development of new hardware and new technologies in wireless networks makes it practical for small devices with sensing abilities to be connected into sensor networks. A considerable amount of attention and research has been devoted in recent years to the deployment of mobile sensing for use in data gathering, healthcare, collaborative information processing and environmental monitoring. By collaborating and exchanging information, sensors ensure energy efficiency. Theoretical research and practical experience have shown that optimal collaboration data gathering strategy among mobile sensor nodes can significantly balance the energy consumption of cluster heads and prolong network lifetime. In the mobile environment, sensor networks are robust and the topology may vary with the moving node. Energy consumption and energy balancing is still a challenging issue, especially in heterogeneous or mobile environments.

Due to the fact that the sensor nodes are usually powered by batteries, the finite power supply makes maintaining the accessibility of the sensor nodes for long-term monitoring tasks a challenge. Therefore, energy efficiency is a key consideration in the development of wireless sensor networks (WSNs), especially in the oil and gas industry field. The rapid development of wireless sensor networks in recent years makes them a feasible solution for the oil and gas industry. WSNs can be remotely used to monitor pipeline, natural gas leaks, and to supply aids to oil and gas industries for platform safety improvement, operation optimization, problem prevention, error tolerance, and operating cost reduction [1,2]. Pipeline leak accidents happen frequently due to aging effect of the pipelines, corrosion and other natural or man-made reasons. This not only causes huge economic losses, but also leads to severe damage to the ecological environment, threatening the lives of people. Therefore, establishing a pipeline leak monitoring system has important practical significance. In the past decades, wireless sensor networks have shown their great potential in reliable monitoring applications in the oil and gas industry. This paper designs a pipeline inspection and oil leakage monitoring system based on wireless sensor network technology. A lot of efforts have been devoted to maximizing the lifetime of WSNs [3,4,5,6,7].

Traditional routing protocols include the one-hop protocol, multi-hop protocol and cluster-based protocol. One-hop protocol has the “limited transmission range” drawback, while the multi-hop protocol has the “serious delay” problem. Cluster-based protocol adopts a hierarchical architecture, in which data is first aggregated in the cluster-head and then sent to the base station. Compared with the one-hop and multi-hop protocols, it is more suitable for long-range and time-critical applications. However, in static clustering, the unlucky cluster-heads would die quickly, and end the useful lifetime of all nodes belonging to those clusters. By using the randomized configuration of the high-energy cluster head, the low-energy adaptive clustering hierarchy (LEACH) algorithm can aid in prolonging network lifetime greatly [7,8]. However, its performance is limited when it involves mobile sensors [9].

One of the key issues in WSNs, besides prolonging network lifetime, is to realize energy-efficient collaborations among sensor nodes in order to compensate for limited sensing, and limited processing capabilities in these nodes. The main goal of this paper is to propose an efficient collaborative strategy for oil exploration, extraction, transportation and storage, with a distributed sensor network. This paper considers that nodes locally exchange coded informative data before transmitting the combined data towards a remote fusion center in a collaborative wireless sensor network. Moreover, this paper will elaborate the potential benefits of sensor collaboration, and discuss the challenging issues and future research opportunities in this area, focusing on distributed sensing and collaborative information processing through WSNs.

We propose a collaborative weighted clustering algorithm (CWCA) in this paper. It uses a weighted sum of several metrics such as the degree, the Euclidean distance, the relative mobility, and the lifetime of a node as a cluster head [9]. The advantage of such an algorithm is that the weight parameters can be adjusted according to the mobile wireless sensor networks requirements. The flexibility in changing the weight factors is helpful for the algorithm’s application in various types of networks. Some simulation experiments have been carried out to evaluate the performance of our proposed algorithm. Both the static sensors and mobile sensors are taken into account. The experimental results show that the LEACH and CWCA can achieve a longer network lifetime than the static clustering algorithm. Although the difference between performances of these two algorithms was limited, with only static sensors in the application that evolves mobile sensors, the CWCA would behave better.

The rest of this paper is organized as follows: various effective collaborative strategies are reviewed in detail in WSNs. Section 3 introduces the background of this research, including the basic idea of application scenario and traditional WSNs routing protocols. Section 4 proposes a collaborative weighted clustering protocol. Section 5 presents illustrative simulation results evaluating the performance of the proposed algorithm for some system configurations and network topologies. Section 6 finally summarizes the research work of the full paper, and discusses future research challenges and opportunities.

## 2. Related Works

In recent years, collaborative information processing in sensor networks has become a very attractive research field. In this section, we provide a brief review of the various existing efficient collaborative strategies that mainly consider the energy efficiency of nodes in WSNs.

Lots of researchers have studied various aspects of the collaboration strategy in WSNs. For example: Agre and Clare [10] proposed a layered architecture for the distributed sensor networks that integrated the cooperation into autonomy. They investigated the architectural aspects of the cooperative signal processing, and emphasized the autonomy of the sensor nodes at the low level. The complex sensor network improves its collective behavior by moving to higher layers and the individual sensor node improves its autonomy by moving to the lower layers.

In order to compress sensor data from individual nodes to obtain minimal inter-sensor communication, Pradhan *et al.* [11] proposed a collaboration strategy that allows minimizing the amount of inter-node communication while preserving the resolution of the data gathered. In order to establish and maintain connectivity in WSNs, Sohrabi *et al.* [12] proposed a number of algorithms that aimed at the self-organization of the wireless sensor networks, exploiting the low mobility and abundant bandwidth, while coping with the severe energy constraint and the requirement of network scalability.

Chu *et al.* presented data-querying and routing approaches in WSNs with an objective of energy efficiency [13]. The proposed approach relied on two key ideas: information driven sensor querying to optimize sensor selection and constrained anisotropic diffusion routing to direct data routing and incrementally combine sensor measurements to minimize an overall cost function. Park *et al.* [14] proposed a protocol for distributed and collaborative multi-hop routing of wireless sensor packets for transmitting plant information throughout the network. The proposed protocol aimed to optimize the routing sequences within the WSNs and helped improve the robustness and reliability of the network. A group of algorithms for wireless collaborative sensing and actuation were given in [15]. The proposed algorithms were validated for optimal control of lighting or temperature in industrial workspaces.

Chen *et al.* [16] proposed a distributed collaborative estimation and control approach for wireless sensor and actuator networks, which accounted for packet loss during wireless communications in noisy industrial environments. The proposed approach exploited the collaborations between actuators and sensors based on a locally collaborative control algorithm. A group connectivity model was proposed in [17] for deployment of wireless sensors in a collaborative operation scheme suitable for harsh industrial environments where running wires were less practical and also prohibitively expensive. The neighboring sensors were connected actually by the common keys in the group connectivity model, which can be used for security enhancement such as encryption purposes within the industrial networks.

The applicability of data fusion for fault diagnosis in collaborative wireless sensor networks was explored in [18]. The authors focused primarily on the use of collaborative wireless sensor networks to monitor the condition and performance of the industrial machines and plants. Naqvi *et al.* proposed a novel collaborative communication system with imperfect phase synchronization that included the influence of noise and Rayleigh fading [19]. Experimental results had shown that the proposed collaborative communication system was energy efficient and can be implemented in sensor networks with a power requirement approximately *N* times less than that with the single-input single-output communication, as far as a specific transmission range was concerned.

In [20], Snoussi *et al.* proposed an efficient collaborative strategy for online change detection of a distributed sensor network. The collaborative strategy ensured the efficiency and the robustness of the data processing, while limiting the required communication bandwidth. There were many parameters that affected the impact of cooperation on sensor network lifetime such as number of domains, node density, network area, propagation environment, network topology, and base station deployment [21]. Bicakci *et al.* investigated cooperation strategies within a linear programming framework to prolong sensor network’s lifetime for use in multi-domain wireless sensor networks.

In [22], Pan *et al.* proposed a novel collaborative signal and information processing (CSIP) algorithm based on virtual fields excited by sensor nodes in wireless heterogeneous sensor networks. Experimental results demonstrated that the proposed approach can reduce energy consumption in sensor nodes, meanwhile, the information gain efficiency and the network lifetime were also increased. Wireless sensor networks have been explored for adoption to improve the performance of the centralized and cable-based structural health monitoring systems. Wu *et al.* designed a multi-agent system and evaluated it for the adoption of collaborative wireless sensor networks in large structure health monitoring [23].

Kalpakis and Tang proposed a method utilizing the measurement results of co-occurrence to identify data redundancy, and a novel collaborative data gathering approach utilizing the co-occurrence to offer a trade-off between the communication costs and communication errors of data gathering for estimating the sensor measurements at the base station [24]. The distributed node wakeup of wireless sensor networks was in the scope of collaborative optimization. Wei and Yong proposed an effective clustering protocol for information potential fields navigation in wireless *ad-hoc* sensor networks [25]. The proposed distributed infectious disease model is utilized to derive the collaborative sensor wakeup method. Visual target tracking which is always performed in single-view system is challenging in complex situations. Wang and Wang presented a distributed multi-view tracking system using collaborative signal processing for distributed wireless sensor networks [26].

The potentiality of WSN-controlled agricultural activities and different environmental compartments for integrated water quality management had been presented and the limitations of WSNs in agriculture and water quality monitoring have been identified. Zia *et al.* proposed a case of collaborative networks at a catchment scale to enable the cooperation among individually networked activities for integrated water quality monitoring, control and management [27]. Fan and Yuan proposed an improved lower bound for Bayesian network based on clustering protocols [28].

## 3. Background

### 3.1. Traditional Routing Protocols

Traditional routing protocols can be classified into three categories: one-hop protocols, multi-hop protocols and cluster-based protocols [29].

**Figure 1:**
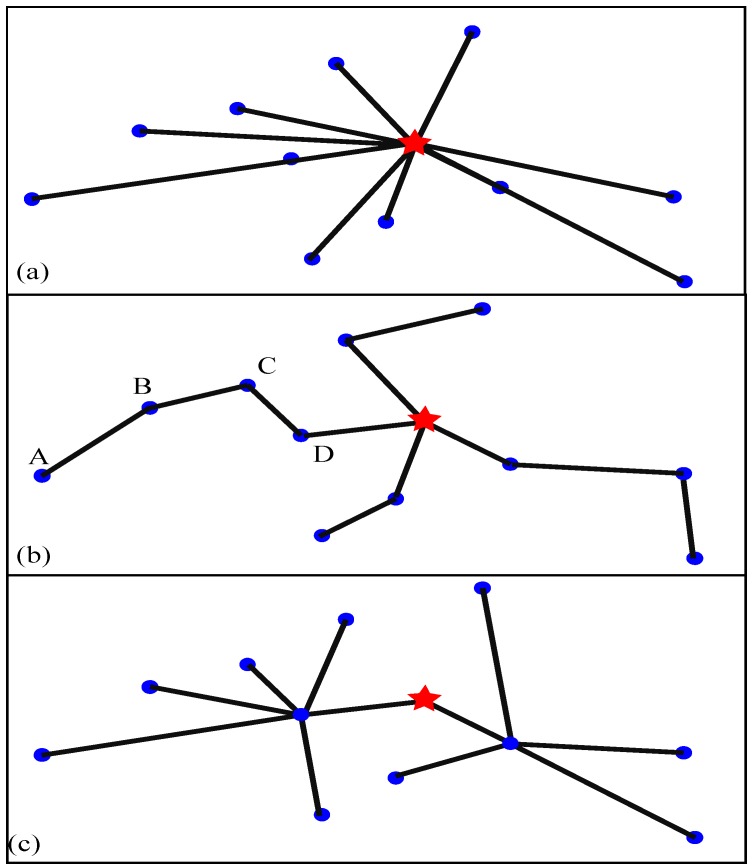
Traditional wireless network routing protocols: (**a**) One-hop protocol; (**b**) Multi-hop protocol; and (**c**) Cluster-based protocol.

One-hop protocols, also known as direct communication protocols, mean that each sensor node in the network transmits the message to the base station. Figure 1a shows a one-hop protocol. They are the simplest protocol models in wireless networks [30]. However, they have several disadvantages. First, since the sensor nodes have limited transmission range, nodes far from the base station may not have enough power to reach it [31]. Second, even if the sensors are close to the base station, the network might be overly dense so the network efficiency would be seriously degraded by collisions [32]. Third, sensor nodes located in the same region may transmit similar results to the base station, which means WSNs exhibit inherent redundancy in their transmissions [33].

Multi-hop protocol are a more practical approach for dealing with the above problems. As shown in Figure 1b, node *A* which is far from the base station does not transmit the information to the base station directly, but rather forwards its data to one of its neighbors (node *B*) which is closer to the base station. Then, node B will in turn send data to its neighbor *C* that is closer to the base station. In this way, the data will finally travel from the source node *A* to the base station by hopping from one to another. Since the path between two neighbor-nodes is much shorter, and consumes less energy, all the nodes in the network are reachable in the end [34,35]. Meanwhile, data aggregation can be used on the travel path, which will reduce the inherent redundancy of the WSNs and further reduce the power consumption. The main drawback of multi-hop protocols is that data experiences serious delays. Four steps are required to transmit data from node *A* to the base station. By the time it reaches the base station, it may already be obsolete [36,37,38,39,40].

Cluster-based protocols are a hierarchical approach that breaks the network into clustered layers. For example, there are two clusters in Figure 1c. In each cluster, there is a cluster head that has the responsibility of routing from the cluster to the base station. Compared with multi-hop protocols, this moves the data faster to the base station and reduces the transmission delays greatly [41]. Only two transmission steps at most are required for transmitting data from one node to the base station. Clustering provides inherent optimization capabilities such as the possibility of data aggregation at the cluster heads [42,43,44,45]. Further, in cluster-based models only cluster-heads perform data aggregation while each intermediate node performs data aggregation in the multi-hop model. The wireless data transmission is completed by the cluster-based method, which overcomes the problem of low detection accuracy caused by the large span of pipeline space. At the same time, as they also have the ability to deal with local signals, sensor nodes using data fusion technology can complete on their own a lot of signal processing, effectively reducing the volume of data transmission, so the decisions havve higher reliability and flexibility, improving to a great extent the monitoring system running speed. Therefore, cluster-based models are more suitable for time-critical applications than multi-hop models. They have low power consumption, and can accomplish the practical functions of real-time monitoring and alarming.

### 3.2. Application Scenario

**Figure 2:**
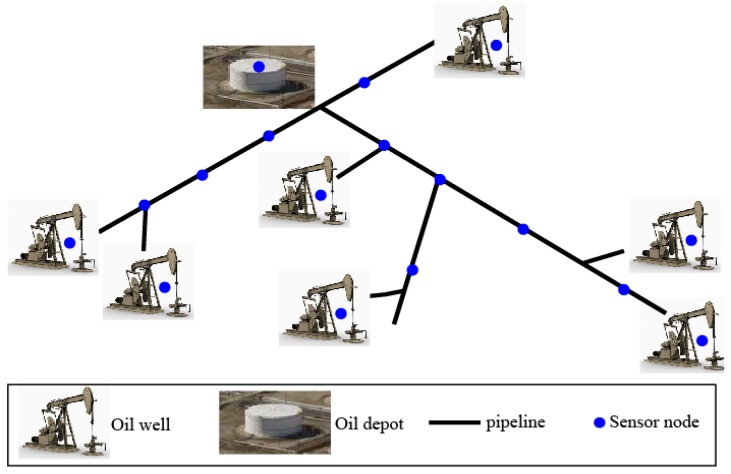
Application scenario.

The oil and gas industry involves processes such as exploration, extraction, storage and marketing to finally obtain petroleum products [46]. The focus of this paper is on gathering and transmitting the wireless information during oil exploration, extraction, transportation and storage. A scenario of such an application is illustrated in Figure 2. It is shown that sensor nodes can be installed on different facilities to collect different signals. With the help of these sensor nodes, the following tasks can be achieved.

Firstly, the sensors may aid in monitoring the production process, to either detect fault issues or to enhance production [47,48]. For example, the sensor node on a pump-jack of an oil well can gather the electric parameters, temperature, pressure and payload of this pump-jack. Such parameters are important in the fault detection of a pump-jack. By diagnosing and solving the faults in time, the reduction of oil production can be prevented.

Secondly, the transportation system for oil or natural gas consists of a complex network of pipelines, and is designed to transport oil or natural gas quickly and efficiently from its origin to areas of high demand. Sensors along the pipeline can be used for state-monitoring of the transportation process. By this means, pipeline leakages can be detected once they happen. The leakage points can be further located with the signals gathered from different sensors along the pipeline. In addition, in order to prevent oil/gas leakage in advance, it is highly desirable to perform pipeline inspection regularly. Mobile sensors are typically required for such situations.

Finally, one of the key imperatives of the oil storage process is that the safety of the oil depot must be guaranteed. By monitoring the equipment condition and the reservoir real-time states, disasters such as explosions and fires can be prevented. The technical requirements for the deployment of WSNs in oil and gas industries are typically more critical than for those in other fields. According to recent reports [49,50], these technical requirements include:

(1) Operate in hostile areas where environmental and platform conditions may be very harsh. Because oil facilities are often installed far away from the cities and towns, it is very possible that no cellular mobile communication services are available.
(2) Meet the requirement of temporary sensor installation.
(3) Reduce maintenance difficulty with limited battery consumption.

### 3.3. Virtual Grid Margin Optimization

According to the assumptions of network model, *N* nodes are distributed randomly in a square area of length *l*. The area of interest is partitioned into grids of equal size, and the nodes within each grid are self-organized into a cluster. The optimization of virtual grid margins impacts greatly the partition of clusters and the whole network’s energy consumption. Consider two extreme cases: one is given such that the distance between two neighboring grids equals the perimeter of the entire region, which is to say, all nodes interact with the cluster head in a single-hop communication manner. In this case, the energy of member nodes would be exhausted rapidly due to the large communication radius of the network. The other is given such that the distance between two neighboring grids is excessively small, in this case, the coverage range of a single cluster would become excessively small, and the maintenance cost for each cluster would become excessively high. Moreover, a small distance between cluster heads may lead to disturbances and reduce the validity of data transmission in the network.

According to the derivation of the overall energy consumption in the network, the optimal cluster number can be calculated, and the grid margin can be optimized based on this calculated result. In the process of selecting the cluster head, we assume that the temporary cluster head participating competition is denoted by *ρ* + Δ*ρ*, the transmission radius of the broadcast message is denoted by *R*, and the length of the message is denoted by *l*_0_, so that the energy consumed by the network can be calculated by:

(1) $$\begin{array}{l} E_{comp}=(\rho +\Delta \rho )[E_{CH-broad}+(N-1)E_{non-CH-broad}]\\ =(\rho +\Delta \rho )(l_{0}E_{elec}+l_{0}\varepsilon_{mp}R^{4})+(N-1)(\rho +\Delta \rho )l_{0}E_{elec} \end{array}$$

In which, *E_CH-broad_* is the energy consumption of broadcast message when the node is a cluster head, and *E_non-CH-broad_* is the energy consumption of broadcast message when the node is a non-cluster head.

Since the transmission radius of the broadcast message is usually greater than the threshold distance *d*_0_ in competition, the energy consumption of the power amplifier should be described by the multiple-path decline signal channel model. During the process of cluster head selection, the cluster head node receives the message from other member nodes. The energy consumed in this process is given as follows:

(2) $$\begin{array}{l} E_{form}=\rho E_{CH-join}+(N-\rho )E_{non-CH-join}\\ =\rho [l_{1}E_{elec}(\frac{N}{\rho }-1)]+(N-\rho )[l_{1}E_{elec}+l_{1}\varepsilon_{fs}d_{toCH}^{2}] \end{array}$$

In which, *E_CH-join_* is the energy consumption of a cluster head node that receives the message from other member nodes, and *E_non-CH-broad_* is the energy consumption of a non-cluster head node receives the message from other member nodes.

After the completion of cluster head selection, the cluster head receives the collected data from its member nodes in the steady running stage. Let *l* denote the bit size of the data packet transmitted in one cycle. The cluster head is responsible for sending data to the base station after the data is collected from the member nodes at the cluster head. In order to guarantee the integrity of all sensor nodes’ data and reduce the cost of data recovery, the cluster head should not execute any data fusion task, and the cluster head nodes’ data is acquired by utilizing mobile sinks, thus the energy consumed in this stage can be written as:

(3) $$\begin{array}{c} E_{tran}=(N-\rho )E_{non-CH-tran}+\rho (E_{CH-rec}+E_{tran-sink})\\ =(N-\rho )[lE_{elec}+l\varepsilon_{fs}d_{toCH}^{2}]+\rho [lE_{elec}(\frac{N-\rho }{\rho })+lE_{elec}+l\varepsilon_{mp}d_{toBS}^{4}] \end{array}$$

In which, *E_tran-sink_* is the energy consumption of the cluster head node transmits the message to the mobile sinks in one cycle. The total energy consumed by the network during one cycle is given as:

(4) $$E_{total}=E_{form}+E_{comp}+E_{tran}$$

Taking into account the length of the control class message in the network is much less than the length of the actual packet, we assume that *l*_0_ = *l*_1_ = (1/*δ*)*l*, δ>>1. In the cluster head selection stage, the energy consumed by a temporary cluster head is far less than that consumed by the whole network broadcasting a short message in one cycle. To obtain a simplified model for the energy consumption calculation, the calculation equation mentioned above could be further transformed and reduced as:

(5) $$E_{total}=[2(N-\rho )+\frac{(2N-1)\rho }{\delta }]lE_{elec}+(N-\rho )(1+\frac{1}{\delta })l\varepsilon_{fs}d_{toCH}^{2}+\rho l\varepsilon_{mp}d_{toBS}^{4}+\frac{\rho }{\delta }l\varepsilon_{mp}R^{4}$$

We assume that the probability density of the nodes in the cluster is denoted by *ρ*(*x*,*y*), and the cluster head locates at the middle of the cluster, then the expected value of the square of the distance between ordinary nodes and cluster head can be calculated by:

(6) $$E[d_{toCH}^{2}]=\iint d_{toCH}^{2}\rho (x,y)dxdy=\iint (x^{2}+y^{2})\rho (x,y)dxdy=\iint r^{2}\rho (r,\theta )rdrd\theta \;=\rho \int_{\theta =0}^{2\pi }\int_{r=0}^{L/\sqrt{\pi k}}r^{3}drd\theta =\frac{\rho L^{4}}{2\pi k^{2}}=\frac{L^{2}}{2\pi k}$$

Substituting Equation (6) into Equation (7), and letting its first-order derivative about k be equal to zero, then the number of optimal clusters can be obtained as:

(7) $$\hat{\rho }=\frac{\sqrt{N}}{\sqrt{2\pi }}\times \sqrt{\frac{\delta \varepsilon_{fs}}{(N-\delta )\varepsilon_{mp}}}\times \frac{LR^{2}}{d_{toBS}^{2}}$$

It can be observed from the Equation (9) that when the number of clusters equals the optimal clustering number $\hat{\rho }$, the variable *T_total_* can take its minimum value, and the energy consumption of the whole network would be optimized in the same time.

According to Equation (9), the number of optimal cluster as well as the total number of the grids partitioned on the monitoring area can be obtained, respectively. Since the shape of each grid is made up of a square, the total number of the grids can be calculated as:

(8) $$Gr_{num}=\mathrm{int}(\sqrt{\hat{\rho }})$$

To obtain a full coverage of the area of interest and avoid losses of acquired data, grid margin should satisfy the condition *Gr_num_* × *D*^2^ ≥ *R*^2^, and obtain:

(9) $$D=\left\lceil \frac{R^{2}}{Gr_{num}}\right\rceil$$

Once the area of interest is partitioned completely by virtual grids, each grid area would have an unique indexing number, denoted by [*Gr_x_*,*Gr_y_*]. Each sensor node is assigned a coordinate (*x_i_,y_i_*) in its initialization stage, and the grid that belongs to each node can be calculated by the following equation:

(10) $$\{\begin{array}{c} Gr_{x}^{i}=\left\lceil \frac{x_{i}-x_{0}}{D}\right\rceil \\ Gr_{y}^{i}=\left\lceil \frac{y_{i}-y_{0}}{D}\right\rceil \end{array}\;$$

where *x*_0_*,y*_0_ represent the initial values of the horizontal and the vertical distances between the base station and the area of interest, respectively. Nodes in the network would fail to perform due to energy dissipation. When the number of failed nodes reaches a specified threshold value, the base station would recalculate the margins of the virtual grids, and then send a broadcast message. In this manner, the cluster would be reconstructed.

## 4. The Proposed Collaborative Weighted Clustering Protocol

In this section, we consider a network of wireless sensor nodes distributed in a region. Each node has a limited energy supply and generates information that should be transmitted to a base station [51]. We further assume that oil depot is not only the destination of the oil transportation, but also the sink of the collected information. In other words, the oil depot also serves as the base station in the WSN. All the signals collected by the sensor nodes will be transmitted to here wirelessly.

### 4.1. System Optimal Model and Problem Formulation

To calculate the power dissipation in the WSNs, the first-order radio model used in [52] has been applied. In this model, to transmit/receive a *k*-bit message over a distance *d*, the energy consumption is calculated by the following equations:

(11) $$\left\{ \begin{array}{l} E_{Tx}=E_{elec}\times k+\varepsilon_{amp}\times k\times d^{2}\\ E_{Rx}=E_{elec}\times k \end{array}\right.$$

where *E_elec_* = 50 nJ/bit is a radio dissipate to run the transmitter or receiver circuitry, and *ε_amp_* = 100 pJ/bit/m^2^ is the transmission coefficient for achieving an acceptable *E_b_*/*N*_0_. Based on the first-order radio model above, the following assumptions are made to simplify the analysis.

Firstly, most sensor nodes cannot adjust their transmission power continuously and normally have only a few discrete power levels, which enable data transmissions with different distances [53]. Thus, *d* is also discredited in this research. This is realized by dividing the whole of application scenario into *n* × *m* grids. Each sensor node is located at the grid that is the nearest one to its real position.

Secondly, it is assumed that the radio channel is symmetric. More specifically, the energy required to transmit a message from node *A* to node *B* equals the energy required to send the same message back.

Thirdly, it should also be noted that receiving a message is also an energy-cost operation. The protocols should thus try to minimize not only the transmission distance but also the number of transmission and receiving operations for each message [7]. Since there are always data redundancy in the system and data aggregation is usually necessary.

### 4.2. Dynamic Clustering Protocols

It is obvious that if the cluster heads are fixed during the operation, the unlucky cluster heads would die quickly and end the limited lifetime of all nodes that belong to those clusters [54]. Thus, it is recommended to employ a dynamic clustering protocol, which has a mechanism for changing the cluster heads in each round to distribute the energy load evenly over the whole network.

In this section, the basic operation steps and the selection algorithm of the cluster heads are introduced. A dynamic clustering operation is broken into several rounds. Each round begins with a cluster set-up phase, followed by a steady-transmission phase. The steady transmission phase is longer compared with the cluster set-up phase for minimizing overhead.

In the cluster set-up phase, each node decides whether or not to become a cluster head for the current round. In this procedure, the popular algorithm named LEACH has been widely used in the past, in which a random number between 0 and 1 is chosen by a sensor node *n*. If the number is less than a threshold *T_n_*, the node *n* would become a cluster head for the current round. This threshold is set as:

(12) $$T_{n}=\left\{ \begin{array}{ll} \frac{P}{1-P[r\mathrm{mod}(1/P)]}, & \;n\in G\\ 0, & \;otherwise \end{array}\right.$$

where *p* is the desired percentage of cluster heads, *r* is the current round, and *G* is the set of nodes that have not been cluster heads in the last 1/*p* rounds. During the first round, each node has a probability *p* of becoming a cluster head. Once a given node is selected as the cluster head, it cannot be a cluster head for the next 1/*p* rounds. In this way, the energy load would distribute evenly among the sensors in each cluster and this would not drain the battery of a single sensor, as happens in static clustering.

Once all the cluster heads are determined, they broadcast their statuses to the other sensors in the network. Each sensor node determines the cluster to which wants to belong by choosing the cluster head that requires the minimum communication energy.

In the steady transmission phase, a TDMA scheme is employed in each cluster. More specifically, each cluster head creates a schedule for all the nodes in its cluster. When it comes to the time slot for a given node, this node is allowed to transmit its data to the cluster head, while all the other nodes are required to be silent. This allows the radio components of each non-cluster head node to be turned off at all times except during its own transmit time, thus minimizes the energy dissipation in the individual sensors. Once the cluster head has all the data from the nodes in its cluster, the cluster head would aggregate the data and then transmit the compressed data to the base station. This process may further reduce the power consumption of the whole network.

### 4.3. Collaborative Weighted Clustering Algorithm

It is obvious that the cluster head selection is quite important for the performance of the whole system [55]. In LEACH, the threshold is based on the suggested percentage of cluster heads for the network and the times the node has ever been elected as a cluster head so far [7]. This succeeds in avoiding draining the battery of a single sensor.

However, things get much more complicated when mobile sensors are involved. In order to avoid frequent cluster head changes, it is necessary to select a cluster head that does not move very quickly or change very frequently. When the cluster head moves fast, its member nodes may be out of its communication range. According to the first-order radio model employed in the system, if a cluster contains nodes that are far from each other, the total power consumption will no doubt be high. It is unfortunate that the LEACH algorithm fails to take this distance issue into account. 

**Figure 3:**
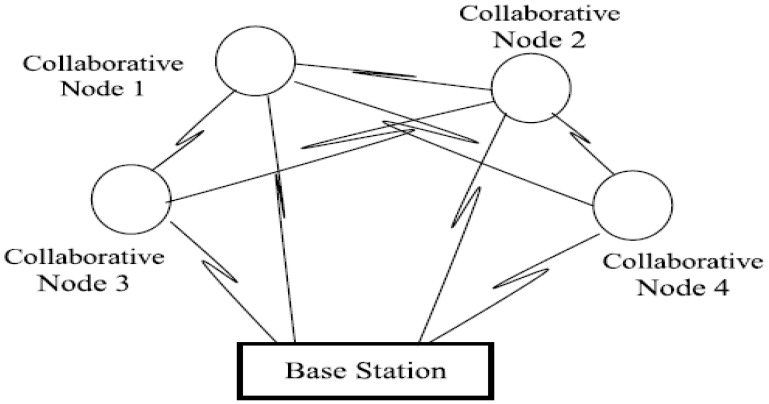
Collaborative nodes transmit the same data to the base station.

In the network, *N* collaborative nodes transfer information to the base station as shown in Figure 3. Let *s*(*t*) be the total amount of information transmission to the base station by the collaborative nodes. In Figure 3, many nodes form a cooperative network to transmit the same data to the base station. In order to achieve power management and connectivity efficiency for mobile sensor nodes, sensor nodes are deployed over a targeted area in a fashion that ensures connectivity. This is done by considering cost efficiency and the event probabilities of the mobile sensor nodes. Sensor nodes ensure the information can be transferred towards the base station by exchanging information within the network. The optimal model of collaborative nodes is defined as:

(13) $$\left\{ \begin{array}{l} z_{t}=P(z_{t}|z_{t+1})\\ x_{t}=A(z_{t})x_{t}+B(z_{t})w_{t+1}\\ y_{t}=C_{m}(z_{t})x_{t}+D_{m}(z_{t})v_{t} \end{array}\right.$$

where $z_{t}$ is the unknown discrete state, $x_{t}$ is the unknown continuous state, and $y_{t}$ is the observations transmitted from the sensor *C_m_* at time *t* to the central processing unit.

To deal with the problem of mobile sensors, a weighted clustering algorithm is employed in this paper. It uses a weighted sum of several metrics, such as the degree, the Euclidean distance, the relative mobility, and the lifetime of a cluster head node. The node with the lowest weight among its neighbors becomes the cluster head. The weight of a node *i* is defined as:

(14) $$weight(i)=\alpha_{1}\times D_{i}+\alpha_{2}\times P_{i}+\alpha_{3}\times M_{i}+\alpha_{4}\times T_{i}$$

where *α*_1_ + *α*_2_ + *α*_3_ + *α*_4_ = 1. *D_i_* is the sum of the Euclidean distances between the node *i* and its neighbors. This is to say:

(15) $$D_{i}=\sum \left\{dist(i,v)\right\}$$

Let *M_i_* denote the average relative mobility of the node. In this study, we assume that *M_i_* = 0 corresponds to static sensor node, while *M_i_* = 1 corresponds to mobile sensor node. *P_n_* is the degree of node *i,* which is defined by finding all the nodes within the transmission range of node *i*. *P_n_* is given as:

(16) $$P_{n}=\left|\sum \left\{dist(n,v)<tx_{range}\right\}-\delta \right|$$

where *δ* is a pre-defined number threshold that each cluster-head can afford. *T_i_* is the lifetime of a cluster head node. It can be defined as follows:

(17) $$T(i)=\frac{1}{1-\sqrt{K_{CH}}\times (r\;mod(\frac{1}{\sqrt{K_{CH}}/n}))}\times (1+\frac{neighbor(i)-\frac{1}{\sqrt{K_{CH}}/n}}{n})$$

In which, *K_CH_* is the employed threshold in LEACH, and *n* is the number of network nodes. The advantage of such an algorithm is that the weight parameters (*α*_1_, *α*_2_, *α*_3_ and *α*_4_) can be adjusted according to the system requirements. The flexibility of changing the weight factors is helpful for the algorithm’s application in various types of networks [9]. The cluster set-up phase of the proposed protocol is a little different from that of the LEACH algorithm. A detailed description is as follows.

Initially, nodes are not associated to any clusters. In order to enable the discovery of neighbors, each node sends an “HELLO” message to its neighbors periodically. When a node receives this message, it updates its neighbor table by adding or updating the information, which includes the address of the node, its coordinates, the number of neighbors and the value of its metric. In this application, this time is fixed at 2 min. Then, the node compares its metric with others. If its value is smaller, it awaits an INVITE message, which is sent by a cluster head inviting other nodes to join its cluster. Otherwise, this node would move to the cluster head state, and send an INVITE message to other nodes.

When a non-cluster head node receives the INVITE message, it stores the information related to the cluster head in its neighbors table, and uses the virtual grid partition method to mark the cluster head node as its parent for routing. Then, it becomes a member of this cluster. On the other hand, if a node at the cluster head state receives this INVITE message, the node compares its metric with the one included in the INVITE message. If it is better, the sender would be considered as its parent, and the node would shift to the member state.

## 5. Simulations and Results

In this section, in order to test the validity of the proposed protocol, MATLAB simulations are carried out with a random network partially shown in Figure 2. In our proposed collaborative communication system, sensor nodes transmit the same data towards the base station. We assume that there are two types of sensors employed in the application scenario. More specifically, they are static sensors and mobile sensors, respectively. It is further assumed that all the static sensors are fixed on the pump-jacks, and sense the payload parameters of the pump-jacks at a fixed rate, while having data sent to the base station periodically. On the other hand, mobile sensors can move along the oil pipelines for temporary use (e.g., pipeline inspection [47] and leakage monitoring [48]). Simulations are carried out at different node deployment scenarios. Table 1 gives some important parameters used in our simulation.

### 5.1. Normal Production Monitoring

**Figure 4:**
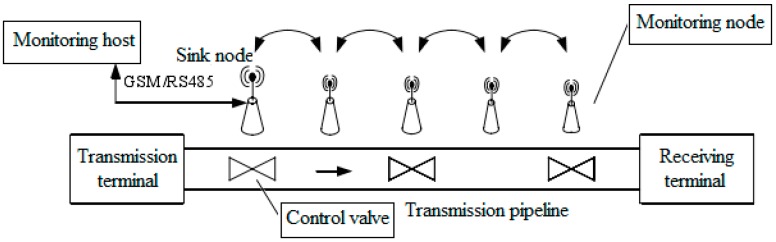
The structure of a pipeline inspection and oil leakage monitoring system.

At first, normal production monitoring simulation was performed. In this case, only the static sensors are analyzed in the simulation. The initial amount of sensor nodes was 100 in total, and the parameters of CWCA were chosen to be *α*_1_ = 0.2, *α*_2_ = 0.2, *α*_3_ = 0.3 and *α*_4_ = 0.1, respectively. Figure 4 shows the overall structure of pipeline inspection and oil leakage monitoring system based on a wireless sensor network. The monitoring system mainly consists of three parts: monitoring nodes, sink node and control valve, which is responsible for data collection and control management of the wireless sensor network.

**Figure 5:**
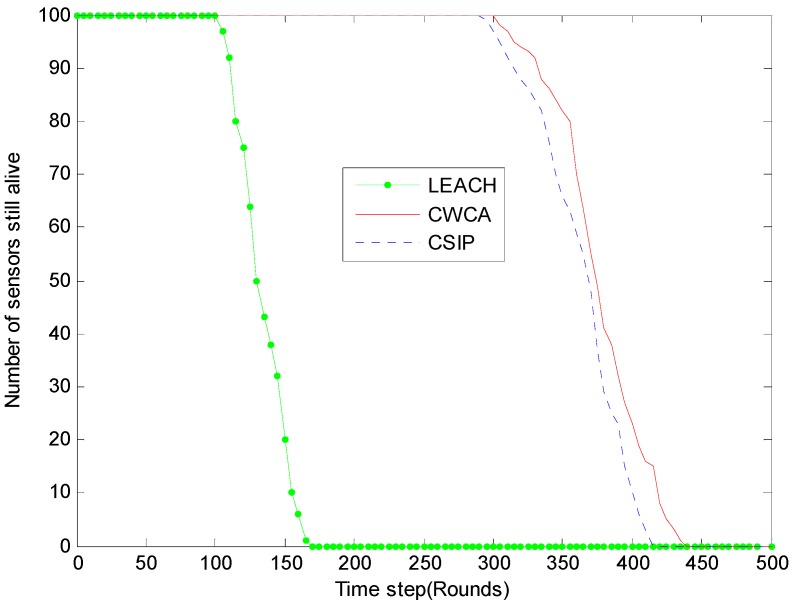
Number of sensors still alive for different time steps in normal production monitoring.

Figure 5 shows a comparison of lifetimes using CWCA, *versus* LEACH and CSIP [22], where the cluster heads and associated clusters are chosen initially and remain fixed and data fusion is performed at the cluster heads. 

We examine the possibility of exploiting the collaborative diversity among the nodes to achieve a large transmission range. When using a LEACH protocol, the cluster-head nodes would die quickly due to the heavy transmission tasks they undertake. Although the non-cluster-head nodes are still alive at that time, the cluster was in fact dead since there is no way to getting their data to the base station. As soon as the last cluster head died, the lifetime of the whole network would come to an end. Meanwhile, due to the fact the cluster heads are configured to be distributed evenly in the network, they die almost at the same time. The difference between the round that the first node dies and the round that the last node dies is no more than 10, which results in the sharp downward curve of static clustering shown in Figure 5.

The advantage of using dynamic clustering (CWCA and CSIP) *versus* using LEACH can be clearly seen in the simulation results. The useful lifetime of dynamic clustering is almost six-times longer compared with that of the static approach. Besides, the dropdown of the dynamic curves is much slower. The performance difference between CWCA and CSIP is not obvious enough in this simulation. We ran similar experiments with different weight parameters and the results remained almost the same.

**Figure 6:**
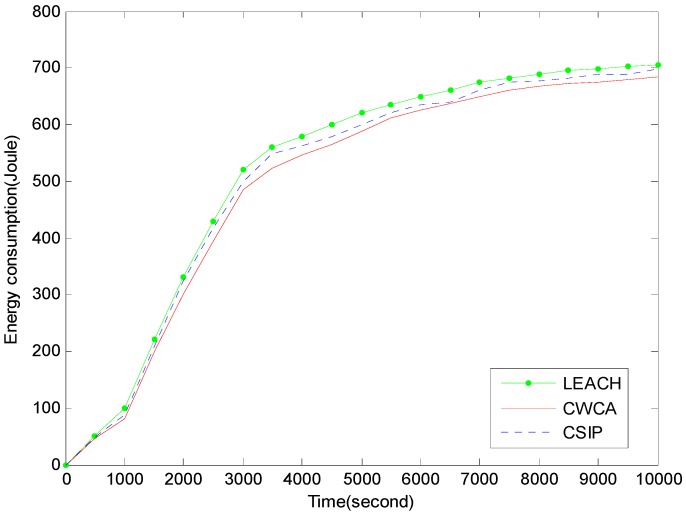
Energy consumption *versus* simulation time in normal production monitoring.

**Figure 7:**
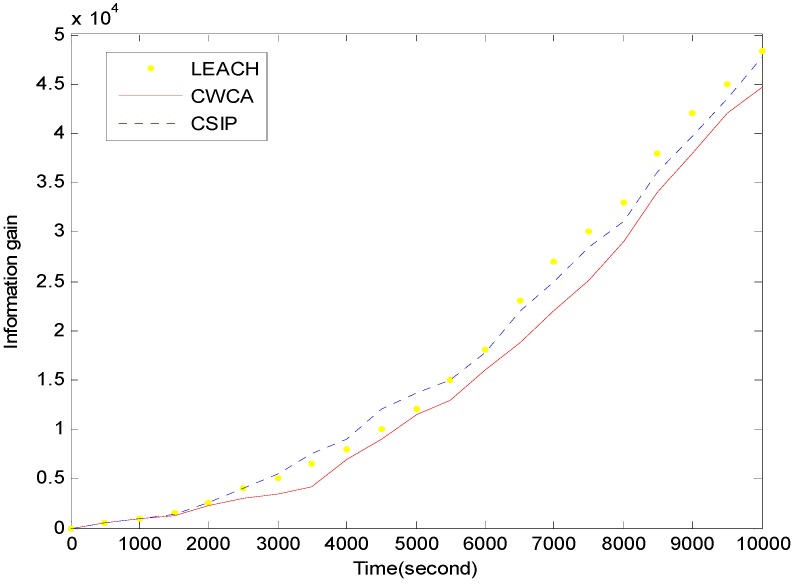
Information gain *versus* simulation time in normal production monitoring.

Figure 6 shows the energy consumption with respect to simulation time in normal production monitoring. Figure 7 shows the information gain with respect to simulation time in normal production monitoring. In CWCA, the sensor nodes communicate with a fusion center during a time frame. We can clearly see that the energy consumption of CWCA is lower than that of other protocols and the information gain is substantial and increases with time.

### 5.2. Pipeline Inspection

In the second simulation, we assumed that a pipeline inspection was carried out. Then, both the static sensors and mobile sensors were taken into account. The amount of static sensors was still 100, and that of the mobile sensor was 1.

This mobile sensor was actually a handheld terminal that can be carried by an inspector. The travel rate of the inspector was set to be 2 grids per round. When the inspector arrives at a grid that has a static sensor node, he would check the sensor node and send the status back to the base station with the help of the mobile sensor. It took about 500 rounds for the inspector to complete the whole pipeline inspection.

**Figure 8:**
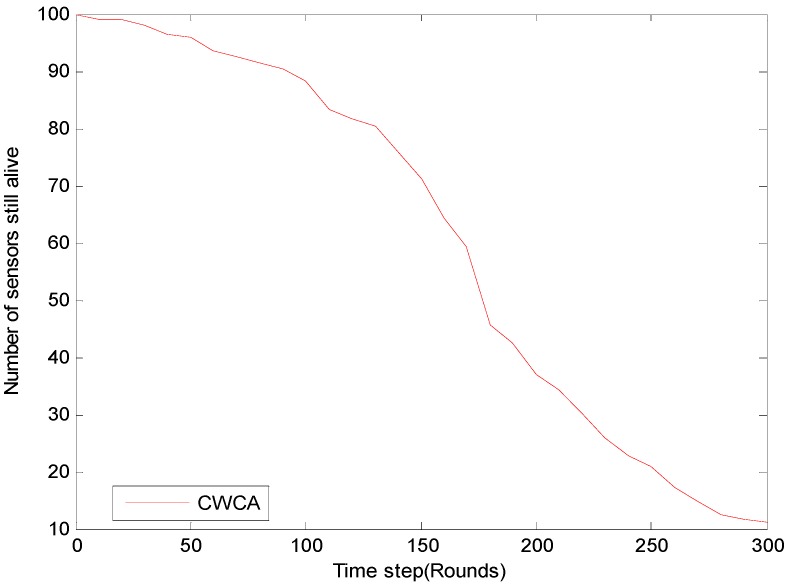
The dump energy of the mobile sensor’s battery when using CWCA.

The dump energy of the mobile sensor’s battery is shown in Figure 8. Each time the mobile sensor was selected as a cluster header, the power consumption would increase accordingly. That’s because not only the data from the mobile sensor, but also the data from the cluster member, was transmitted to the base station during such a procedure. It can be concluded that the less time the mobile sensor has been selected as a cluster header, the more dump energy its battery will preserve till the end of the pipeline inspection.

**Figure 9:**
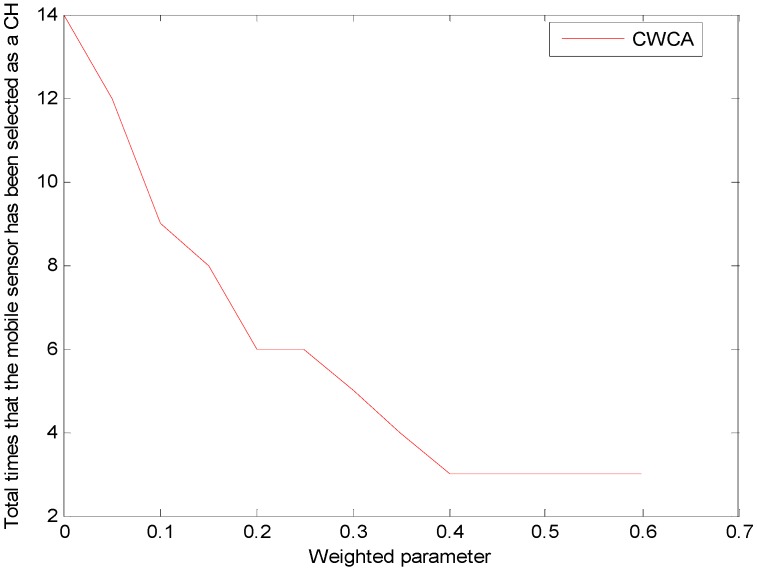
The relationship between *α*_3_ and the total times that a mobile sensor has been selected as a cluster sensor.

In this simulation, we care more about whether this mobile sensor would be selected as a cluster header during the pipeline inspection. The results show that when using LEACH, the mobile sensor would be selected as a cluster header for 13 times. When using CWCA, the times that the mobile sensor is selected as a cluster header would be related to the weight of *α*_3_. Once fixed *α*_1_ = *α*_2_ = 0.2, and changing the weight of *α*_3_ and *α*_4,_ there would be a result such that the smaller *α*_3_ is, the more likely it is the mobile sensor would be selected as a cluster header, as shown in Figure 9.

### 5.3. Oil Leakage Monitoring

In this section, mobile sensors were considered in the simulation. An “event-driven” simulation was implemented. More specifically, it was assumed that a leakage occurs on one of the pipelines, which reduces the oil flow at the end of the pipeline. In order to find the leakage, several sensors were temporarily installed along the pipelines. After the problems were solved, the sensors were removed gradually.

**Figure 10:**
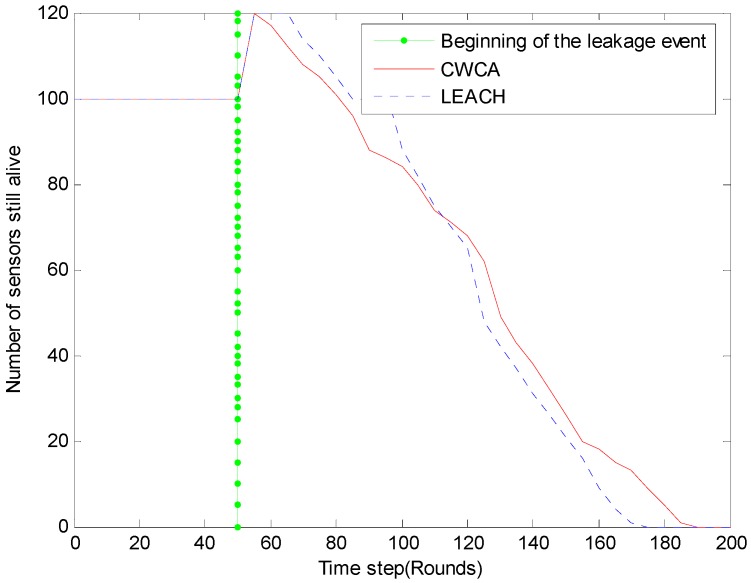
Number of sensors still alive for different time steps in oil leakage monitoring.

**Figure 11:**
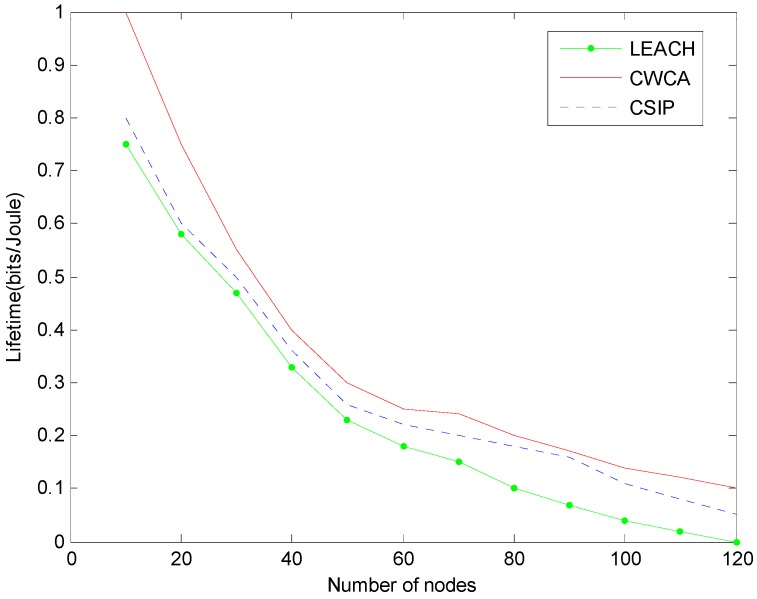
Normalized lifetimes for different network sizes in oil leakage monitoring.

For long-term monitoring applications, the network’s lifetime is especially important. The system’s lifetimes corresponding to CWCA and LEACH are shown in Figure 10. Figure 11 shows normalized network lifetimes for networks with different sizes and cluster-based routing protocols. And the red dash line illustrates the beginning of the leakage event. Once the leakage occurs, the amount of sensor nodes increases from 100 to 120. When using LEACH, the first dead node is discovered soon after the leakage event occurs. It is believed that the instantaneous growth of data traffic is responsible for this. To verify this, we have changed the beginning time at which the leakage event occurs, and a similar phenomenon has been observed.

On the other hand, by using CWCA, it seems that the round in which the first node dies has nothing to do with the beginning time of the leakage. It should also be noted that after the leakage was handled, the amount of sensor nodes decreased to 100 gradually from the 60th rounds. The actual round in which the first node dies occurs at about the 150th round. There is no doubt that the CWCA would behave better than the LEACH when using mobile sensors.

The effect and power test was conducted through the above experiments. The above results show that this model can run well, and the working life of the nodes has been extended efficiently. It is able to conduct real-time pipeline monitoring, accurately reflecting the pipeline’s working state and alarming in a timely way.

## 6. Conclusions

In this paper, we propose a collaborative dynamic cluster-based routing protocol, which has a significant impact on the overall energy dissipation of the wireless sensor network. The experimental results have shown that the proposed protocol is an energy efficient collaborative strategy such that the sensor nodes can communicate with a fusion center during a time frame and produce high power gain. In the future, we will elaborate the potential benefits of sensor collaboration, and focus on distributed sensing and collaborative information processing through WSNs.

## Figures and Tables

**Table 1:** Some important simulation parameters.

No. Item	Parameter Description	Value
1	Communication range	100 × 100
2	Location of the base station	(150, 50)
3	Node number	100
4	Initial energy range	[5,10] Joule
5	Work time period	0.1 s
6	Packet size	500 bytes
